# Biopolymer-Based Composite Hydrogels Embedding Small Silver Nanoparticles for Advanced Antimicrobial Applications: Experimental and Theoretical Insights

**DOI:** 10.3390/polym15163370

**Published:** 2023-08-11

**Authors:** Moises A. Rojas, John Amalraj, Leonardo S. Santos

**Affiliations:** 1 Laboratory of Asymmetric Synthesis, Instituto de Química de Recursos Naturales, Universidad de Talca, Talca 3460000, Chile; morojasn@gmail.com; 2 Laboratory of Materials Science, Instituto de Química de Recursos Naturales, Universidad de Talca, Talca 3460000, Chile

**Keywords:** biopolymers, hydrogels, silver nanoparticles, antibacterial agents, density functional theory

## Abstract

In this work, we report a two-step methodology for the synthesis of small silver nanoparticles embedded into hydrogels based on chitosan (CS) and hydroxypropyl methylcellulose (HPMC) biopolymers. This method uses *d*-glucose as an external green reducing agent and purified water as a solvent, leading to an eco-friendly, cost-effective, and biocompatible process for the synthesis of silver nanocomposite hydrogels. Their characterization comprises ultraviolet-visible spectroscopy, Fourier-transform infrared spectra, differential scanning calorimetry, scanning electron microscopy with energy-dispersive spectroscopy, and transmission electron microscopy assays. Moreover, the structural stability of the hydrogels was investigated through sequential swelling–deswelling cycles. The nanomaterials showed good mechanical properties in terms of their structural stability and revealed prominent antibacterial properties due to the reduced-size particles that promote their use as new advanced antimicrobial agents, an advantage compared to conventional particles in aqueous suspension that lose stability and effectiveness. Finally, theoretical analyses provided insights into the possible interactions, charge transfer, and stabilization process of nanoclusters mediated by the high-electron-density groups belonging to CS and HPMC, revealing their unique structural properties in the preparation of nano-scaled materials.

## 1. Introduction

Metal nanoparticles (NPs) are size- and shape-dependent atom clusters (1–100 nm) that stand out for their large specific surface area to volume ratio [1]. In recent years, the use of NPs has gained importance due to their attractive properties, high efficiency, and low cost, which has led to the use of these materials in a broad range of applications, such as antibacterial, biomedical, pharmaceutical, cosmetic, electronics, and drug delivery systems [2,3]. In the synthesis of reduced-size NPs, nanotechnology plays an integral role. A typical and straightforward method is the chemical reduction of a metal precursor in aqueous solution [4,5]. However, the tendency to agglomerate is a common problem in the synthesis of nanosized particles, negatively impacting their effectiveness for antibacterial applications. In this regard, stabilizers or capping agents are broadly used to inhibit the overgrowth and aggregation of metal atoms during the nucleation of NPs, governing their shape and size [5,6,7]. Typically, stabilizers are derived from either natural or synthetic polymers with chelating ability, acting through intermolecular polymer–metal interactions, especially relevant for biopolymers [8,9].

Among natural polymers, chitosan (CS) and hydroxypropyl methylcellulose (HPMC) are two polysaccharide-type macromolecules that have received significant attention in recent times, due to their biodegradability, biocompatibility, low cost, and interesting structural properties in the preparation of metallic particles [10,11]. In particular, CS is a biodegradable and non-toxic biopolymer derived from the partial (or total) deacetylation of chitin under alkaline conditions [12], composed of β-(1→4)-linked 2-amino-D-glucopyranose and 2-acetamido-D-glucopyranose units. Thus, CS possesses hydroxyl and amino polar groups, which are available for electrostatic interactions and provides a polycationic behavior to its structure [10], offering an attractive dual role as a reductant or stabilizer agent in the synthesis of NPs [13,14]. On the other hand, HPMC is a natural and biodegradable polymer derived from cellulose, which contains several hydroxyl groups and is highly soluble in aqueous solution [11,15]. HPMC has excellent intrinsic properties (e.g., good adhesion, film-forming ability, non-toxicity), and it can also reduce metal ions to metallic NPs in the presence of an external reducing agent [16,17].

Furthermore, biopolymeric materials such as CS and HPMC deserve special attention in the preparation of organic hydrogels a three-dimensional and crosslinked network of polymers needed for antimicrobial applications [18]. During the last decade, advances in this field have been considerable, recognizing hydrogels as suitable matrices to incorporate other materials like metallic NPs to acquire control of their morphology and size [19,20]. Particularly for antibacterial applications, the development of biopolymer-based hybrid hydrogels containing metal NPs arises as an alternative in seeking new materials for the global rise of antibiotics overuse and resistance [21,22,23]. Moreover, the addition of metallic particles into hydrogels can improve their antimicrobial activity [24]. Among metals, silver (Ag) with well-known bactericidal properties [25,26] has been selected to synthesize silver nanoparticles (AgNPs) for advanced antibacterial purposes. At this point, it must be considered that, compared to conventional particles in aqueous suspension, hydrogels embedded with AgNPs provide better binding during antibacterial assays, directly impacting their effectiveness as antimicrobial agents [24]. Nevertheless, despite numerous investigations regarding the use of CS, it is still a challenge to explore its utility along with other polysaccharides like HPMC in the preparation of organic hydrogel matrices and the synthesis of reduced-size AgNPs for further antimicrobial practices [27].

The novelty of the current work Is the fabrication of functional hydrogel–AgNPs materials using biopolymers (CS and HPMC) in environmentally friendly conditions without any organic solvents in order to set a simple protocol to reproduce. These hybrid composites were characterized with experimental techniques and then evaluated for their promising antibacterial applications, against both Gram-negative and Gram-positive bacteria. Additionally, a theoretical approach consisting of polymer–metal complexes was considered to gain insights into the interaction and stabilization mechanisms of selected nanocluster models, resulting in one of a few studies that complement experimental findings with computational analyses.

## 2. Materials and Methods

### 2.1. Chemical Reagents

Chitosan (CS, medium molecular weight, deacetylation degree: 75–85%), hydroxypropyl methylcellulose (HPMC, hydroxylpropoxyl content: ~9%), silver nitrate (AgNO_3_), *N*,*N*’-methylenebisacrylamide (MBA), tetramethylethylenediamine (TMEDA), ammonium persulfate (APS), and *d*-glucose were purchased from Sigma-Aldrich (St. Louis, MO, USA). Acrylamide (AAm) was obtained from Fischer Scientific (Toronto, ON, Canada). All chemicals were used without further purification. The water used in all experiments was purified using a Millipore Milli-Q system. Luria-Bertani broth (LB) and select agar were acquired from Invitrogen (Carlsbad, CA, USA).

### 2.2. Preparation of Polysaccharide-Based Hydrogels

Initially, stock solutions of CS were prepared in falcon tubes by dissolving different amounts of CS (2 mg/mL and 6.92 mg/mL) in 1% acetic acid solution (40 mL). Similarly, in the case of HPMC polymer, stock solutions were dissolved in 40 mL of Milli-Q water (0.5% *w/v* and 3% *w/v*). Later, CS and HPMC hydrogels were prepared in a 50 mL beaker by mixing 1 mL Milli-Q water and 1 mL of the polymer solution; then, the mixture was slowly stirred at ambient temperature before adding and dissolving the monomer (AAm, 1 g). Next, the temperature was increased to 50 °C, and subsequently 1 mL of crosslinker MBA, 1 mL of activator TMEDA, and 1 mL of initiator APS were added. The free-radical polymerization (FRP) was carried out for 30 min until gelation was observed. Finally, hydrogel species were immersed in Milli-Q water to remove all the unreacted materials from the surface and inside the polymer matrix. The water was changed repeatedly every 6 h for 2 days. All gels were dried at ambient temperature for 2–3 days. The composition of the prepared hydrogels is depicted in Table 1 below.

### 2.3. Synthesis of Silver Nanocomposite Hydrogels

Firstly, 100 mg of dry gels were transferred to a 50 mL beaker and equilibrated overnight by adding 25 mL of AgNO_3_ solution (52 mM) at room temperature. During this stage, silver ions are exchanged from the solution to inside the hydrogel network. Beakers were covered with aluminum foil to avoid silver salt decomposition by light. Afterward, the precursor solution was removed, and the gels were wiped off with tissue paper. The hydrogels were transferred to a beaker containing 25 mL of 1% *d*-glucose for 12 h at 60 °C. In this step, Ag^+^ ions are reduced into metallic silver (Ag) and are also stabilized by the internal structure of hydrogels [4,16]. Finally, the obtained CS and HPMC hydrogels embedding silver nanoparticles (termed as CS/AgNPs and HPMC/AgNPs, respectively) were allowed to dry at ambient temperature for the subsequent characterization studies.

### 2.4. Characterization of Nanomaterials

UV-vis measurements of the silver nanocomposites were carried out on a SpectraMax M2 Microplate Reader (Molecular Devices, Sunnyvale, CA, USA), operated in a range of 300–700 nm at a resolution of 1 nm. SoftMax Pro 7.0.3 was used for data acquisition and analysis. All graphs were plotted using Gnuplot 5.2 software [28].

Fourier transform infrared (FTIR) spectroscopy was used to record the spectra of pure CS and HPMC hydrogels and their silver nanocomposite counterparts. The samples were completely dried at 60 °C in an electric oven for 6 h before analysis and then were explored in a wavenumber range of 4000–500 cm^−1^, on a Thermo Nicolet iS5 (Thermo Scientific, Madison, WI, USA) equipped with an iD7 ATR accessory (attenuated total reflectance mode), accumulating 300 scans per sample.

Differential scanning calorimetry (DSC) was performed to evaluate the thermal stability of the CS and HPMC matrices. The denaturation temperature (*T*_d_) of the samples was established from the curve of heat flow vs. temperature using a Q2000 DSC (TA Instruments, New Castle, DE, USA). Heating scans were recorded between 5–200 °C at a scan rate of 10 °C/min under N_2_ flow (20 mL/min).

The internal structure exploration of all prepared hydrogels was assessed using low-temperature cryo-scanning electron microscopy (cryo-SEM) (Tescan VEGA-II XMU VPSEM, Brno, Czech Republic). Samples were firstly frozen at −50 °C and coated with a 5.0 nm-thick carbon film using a low vacuum coater Leica EM-ACE200 (Wetzlar, Germany) and imaged with a secondary emission detector in a JSM-7500F FESEM (JEOL USA Inc., Peabody, MA, USA) operated at 20 kV. Additional energy-dispersive spectroscopy (EDS) (Oxford X-ray INCA-EDS) analysis was also performed to study the elemental composition of the nanostructures. ImageJ 1.52 (National Institute of Health, Bethesda, MD, USA) [29] was used to measure the cavity sizes on the hydrogels.

Transmission electron microscope (TEM) images were collected to obtain insights about the size and morphology of the AgNPs embedded in CS and HPMC hydrogels. The samples were prepared by placing a drop of NPs resuspended from the composite hydrogels on a carbon-coated copper grid and subsequently drying in air, before transferring them to a JEM-2100F FETEM (JEOL Inc., Tokyo, Japan) microscope, operated at an accelerated voltage of 120 kV.

### 2.5. Swelling–Deswelling Studies

The water-absorbing capacity of the prepared hydrogels was evaluated through swelling ratio (*S*_g/g_), according to a previously reported method [30]. Gravimetrically, 100 mg of dried hydrogels were immersed in Milli-Q water at ambient temperature for 24 h to reach swelling equilibrium. Then, samples were accurately re-weighted, using a Mettler Toledo XS105 precision balance (Mississauga, ON, Canada), in order to calculate their ratios by applying Equation (1) below:

(1) $$S_{g/g}=\frac{M_{s}-M_{d}}{M_{d}}$$

where $M_{s}$ = mass of the swollen hydrogel, and $M_{d}$ = initial mass of the dry gel. After deswelling the hydrogels at room temperature for 48 h until a constant weight was obtained, the above process was repeated three times under the same conditions, where the samples were allowed to swell again in water for the evaluation of their mechanical behavior through successive swelling–deswelling cycles. The given values are an average of three individual sample readings.

### 2.6. Antibacterial Activity Assay

The antimicrobial properties of the silver nanocomposite hydrogels were qualitatively assessed through the agar diffusion test, using the standard method reported elsewhere [31,32]. Nutrient agar medium was prepared by mixing LB broth (10 g/500 mL) and select agar (7.5 g/500 mL) in double-distilled water. This agar medium was sterilized in an autoclave prior to use (121 °C for 20 min) and then was passed into glass Petri dishes (15 mL) in a laminar airflow chamber at ambient temperature. The activity was evaluated against the Gram-positive bacteria *Staphylococcus aureus* (ATCC 25923), as well as Gram-negative *Pseudomonas aeruginosa* (PAO1) and *Escherichia coli* (DH5α). Each strain was spread (100 µL) on the solid surface of the medium, containing around 10^6^ CFU/mL. Afterward, swollen hydrogels (20 mg/1 mL) were cut into circular pieces (6.0 mm in diameter) before to being placed into the inoculated Petri dish and then incubated for 24 h at 37 °C. Hydrogels without AgNPs served as controls (pure CS and HPMC gels). At the end, the inhibition zones were measured and photographed.

### 2.7. Computational Details

In the first stage, the initial geometries of selected silver nanoclusters, Ag_n_ (*n* = 2, 4, 6, and 8 atoms), taking as starting conformations those reported by Bonačić [33], as well as 5 monomeric units of CS and HPMC linear polymers (118 and 121 atoms, respectively), were drawn using GaussView 5 software [34] and then were fully optimized at the density functional theory (DFT) level. DFT spin-restricted calculations were performed using the Gaussian 09 computational package [35]. Full geometry optimization of all systems was achieved by means of employing the hybrid exchange-correlation functional B3LYP (Becke’s three-parameter and Lee-Yang-Parr) [36,37] without any symmetry restriction. The triple-zeta 6-311G(d,p) basis set [38] was defined for light atoms (C, H, O, N), along with the relativistic effective core potential LANL2DZ basis set [39] for Ag atoms.

For the potential interactions between the silver nanoclusters and the polymers (CS and HPMC), in the next step, each optimized Ag cluster model was placed at 4 Å of each polymer, then all complexes were re-optimized at the 6-311G(d,p)//LANL2DZ level. The GaussView 5 program was also used to explore the electronic structure in all optimizations.

Hence, the analysis of these interactions was focused on the complexation energies (∆*E_complexation_*), as specified in Equation (2).

(2) $$\Delta E_{complexation}=E_{Ag-CS/HPMC}-\left(E_{Ag}+E_{CS/HPMC}\right)$$

where $E_{Ag-CS/HPMC}$, $E_{Ag}$, and $E_{CS/HPMC}$ are the ground state energies of Ag_n_–CS and Ag_n_–HPMC complexes, Ag_n_ free cluster, and free CS and HPMC polymer models, respectively. Thus, a negative value of $\Delta E_{complexation}$ indicates that the molecular complex is more stable than its separated constituents.

At the end, a natural bond orbital (NBO) analysis [40] was carried out on the complexes, aimed to characterize their charge distribution and intermolecular interactions involved in the stabilization of silver nanoparticles. All graphs were plotted using Gnuplot 5.2 [28].

## 3. Results and Discussion

### 3.1. Preparation of CS and HPMC Hydrogels

**Scheme 1:**
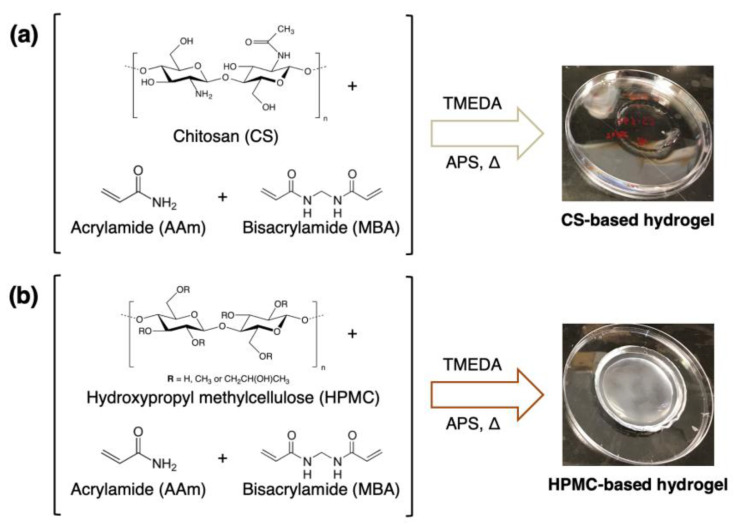
Diagram for the synthesis of pure (**a**) chitosan (CS) and (**b**) hydroxypropyl methylcellulose (HPMC) hydrogels.

Both chitosan (CS) and hydroxypropyl methylcellulose (HPMC) hydrogels were synthesized via the conventional FRP technique, i.e., the polymerization of base polymers in the presence of monomer AAm, cross-linker MBA, along with the initiator pair TMEDA/APS (see Scheme 1). Pure CS hydrogels are tough and visually transparent. On the other side, the HPMC hydrogels have a grayish-opaque coloration, probably due to the viscosity of HPMC powder in aqueous solution and compared to CS gels, they hold a soft and sticky consistency [41]. From previous reports, it’s well documented that the FRP process has many applications, mainly in the preparation of porous polyacrylamide gels as electrophoresis systems [42,43]. However, the inclusion of polysaccharides can modify the properties of the resulting hydrogels, like internal structure, porosity, degradability, absorption, and swelling capacity [44,45].

### 3.2. Synthesis of CS/AgNPs and HPMC/AgNPs Composite Hydrogels

**Scheme 2:**
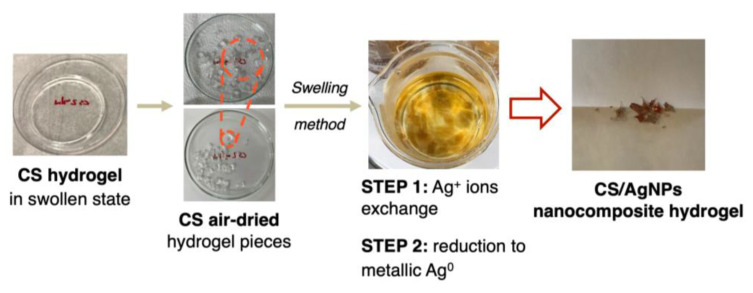
Illustration of the preparation protocol for obtaining CS/AgNPs hydrogels. The same procedure is applied to embed AgNPs into HPMC-based hydrogels.

The preparation of silver nanocomposite hydrogels (CS/AgNPs and HPMC/AgNPs) initially involves the synthesis of pure hydrogels (as represented in Scheme 1), followed by the in situ reduction of precursor salt (AgNO_3_) and subsequent stabilization of AgNPs into the hydrogel network. In general, the chemical reduction of Ag^+^ ions leads to the formation of zerovalent silver (Ag^0^), followed by its agglomeration into metallic clusters, and at the end, silver particles are formed [45]. Scheme 2 shows the proposed two-step protocol for the synthesis of AgNPs using the swelling method, wherein, in the first step, the silver ions are absorbed or anchored by the hydrogel structure. Next, the reduction (second step) is achieved through a green process using *d*-glucose, a cost-effective and environmentally friendly reducing agent that can be easily integrated with natural polymers, to stabilize and protect the metal NPs [46]. Moreover, the reduction process and later stabilization of AgNPs are attributed to the presence of functional groups belonging to CS (amino and hydroxyl) and HPMC hydrogels (hydroxyl). As a result, particles are embedded into internal cavities of the CS/AgNPs and HPMC/AgNPs composite hydrogels, which can be visually confirmed at first instance since the gel changes from transparent to an intense yellowish-brown coloration.

### 3.3. Characterization Results

**Figure 1:**
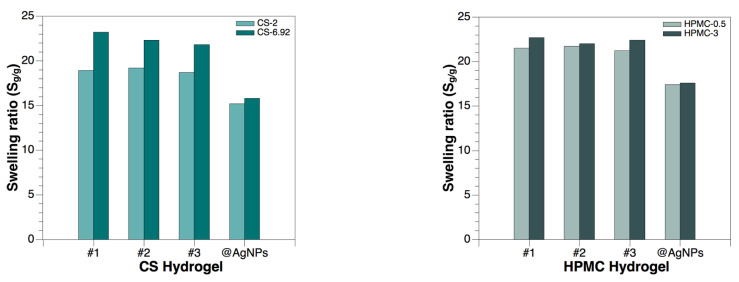
Swelling ratios of the prepared CS (**left**) and HPMC (**right**) hydrogels and their corresponding AgNPs composites (@AgNPs) (# = cycle number for pure hydrogels).

Figure 1 exhibits the swelling–deswelling studies of the formulated hydrogels, pure CS, and HPMC as well as their silver nanocomposites. All these hydrogels retain a higher water uptake capacity compared to the control H-0 (*S_g/g_* = 13.2, as shown in Table 1). Interestingly, prepared CS and HPMC gels maintain a similar swelling tendency during the successive cycles, revealing small variations in their ratios as a sign of stable mechanical behavior. In each case, the order of swelling ratios was found as CS-6.92 > CS-2 and HPMC-3 > HPMC-0.5. As expected, hydrogels at high initial polysaccharide concentration increase the swelling capacity. In particular, HPMC-based hydrogels hold more flexibility causing higher water retention [41]. Moreover, AgNPs are responsible for a moderate decrease in swelling capacity in comparison with their respective pure hydrogels. This data is in good accordance with previous studies, where the inclusion of AgNPs into hydrogels may alter their swelling behavior [32,47,48].

**Figure 2:**
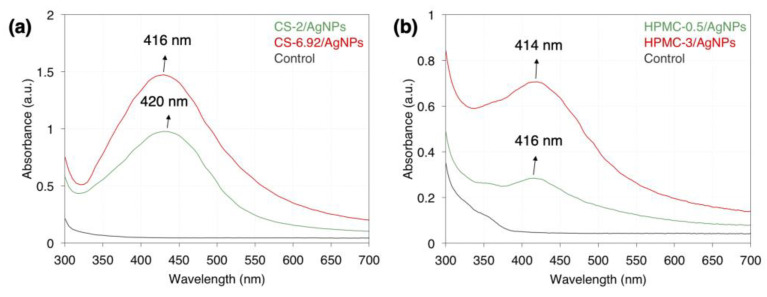
UV-vis spectra of the AgNPs obtained from (**a**) CS and (**b**) HPMC nanocomposites.

Then, the presence of embedded AgNPs in CS and HPMC hydrogels is established by using UV-vis spectroscopy. Figure 2 illustrates the characteristic surface plasmon resonance (SPR) of the AgNPs extracted from the nanocomposite hydrogels. In all prepared composites, a single SPR absorption peak was found, indicating the formation of spherical AgNPs according to Mie’s theory [49]. At higher polymer concentrations for CS/AgNPs composites, increased corresponding peak intensities with blue shifts from 420 to 416 nm for CS-2/AgNPs and CS-6.92/AgNPs, respectively, as can be seen in Figure 2a. This evidence clearly implies that the AgNPs were greatly stabilized by CS into the hydrogel network. However, the increase in intensities is related to an increase in the concentration of AgNPs, not necessarily determining the size of the particles. HPMC nanocomposites follow the same trend, where blue shifts from the range of 416 to 414 nm are observed in the respective cases of HPMC-0.5/AgNPs and HPMC-3/AgNPs (Figure 2b), accounting for the stabilizing role of HPMC in the synthesis of silver NPs [16]. Furthermore, these findings can be complemented by the results obtained from an extensive recent investigation [50], where the authors analyzed the effect of a polysaccharide at various concentrations for aqueous suspensions of AgNPs. In that sense, a decrease of the broadness of the SPR peak indicates that NPs with a narrow size distribution were formed in the CS/AgNPs and HPMC/AgNPs composites.

**Figure 3:**
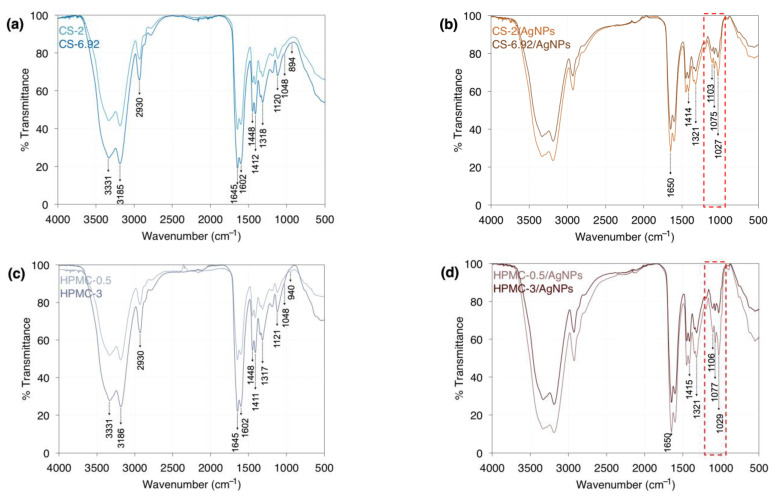
FTIR spectra of the CS, HPMC, CS/AgNPs, and HPMC/AgNPs composite hydrogels. (**a,c**) represents the pure hydrogel composition. The highlighted area in (**b**,**d**) denotes the new peaks that appeared due to the anchoring of silver atoms.

FTIR-ATR analysis is shown in Figure 3 and was conducted to differentiate the composition between pure hydrogel structures and their silver nanocomposites. The spectra of both CS and HPMC gels as well as their CS/AgNPs and HPMC/AgNPs composites are compared according to the absorption peaks associated with the vibration of functional groups ranging from 4000 to 500 cm^−1^. For pure CS hydrogels (Figure 3a), the stretching bands at 3331 cm^−1^ and 3185 cm^−1^ are related to O–H vibration and amide group frequency, respectively. The C–H stretching vibration appears at 2930 cm^−1^, as evidence of CS-MBA crosslinking [51]. Amide I stretching frequency (C=O bond) and N–H bending vibration (amide II) are centered at 1645 cm^−1^ and 1602 cm^−1^. The peaks in the range 1450–1310 cm^−1^ are assigned to C–N stretching vibrations. The asymmetric C–O–C bending vibration of the pyranose ring and the C–OH vibration are visible at 1120 cm^−1^ and 1048 cm^−1^. Finally, a weak band observed at 894 cm^−1^ is attributed to the β-configuration of the D-glucopyranose ring belonging to the CS structure. Compared with CS-6.92, all the peaks in CS-2 spectra are weaker, likely due to the lower concentration of CS inside the hydrogel network. As can be noticed in Figure 3c, some of these transmission bands were also found in the pure HPMC spectra (such as peaks at 3331, 1645, 1448, and 1048 cm^−1^). In addition, the stretching of N–H bonds characteristic of the amide group is visible at 3186 cm^−1^. The absorption band that appeared at 1121 cm^−1^ is attributed to the C–O–C bending of the D-glucopyranose ring, whereas a very weak peak at 940 cm^−1^ was assigned to its β-configuration, confirming the presence of HPMC in the hydrogels [19]. Following the tendency of CS hydrogels, higher intensity peaks are exhibited in the HPMC-3 spectra compared to HPMC-0.5, as expected.

Comparatively, from the FTIR spectra of the AgNPs composites, the main differences with pure hydrogels were found within the fingerprint region around 1700–500 cm^−1^. In particular, for CS/AgNPs hydrogels (Figure 3b), the N–H bending peak at 1645 cm^−1^ is shifted to 1650 cm^−1^. The C–N stretching vibration of the amide III peak is slightly shifted from 1318 cm^−1^ to 1321 cm^−1,^ decreasing its intensity. Additionally, a weak band appeared at 1103 cm^−1^, along with two more intense peaks at 1075 cm^−1^ and 1027 cm^−1^, which evidenced that a covalent Ag–N/Ag–O bonding has occurred [19,31]. Similar wavenumber shifts and new bands were also detected in both HPMC-0.5/AgNPs and HPMC-3/AgNPs spectra (Figure 3d), indicating the formation of a chemical bond between silver and oxygen atoms belonging to HPMC inside the macromolecular structure of these hydrogels [16,52].

Regarding the thermal stability of matrices, the denaturation temperatures extracted from DSC data indicate an improvement in the hydrogel properties after incorporating the AgNPs (see Figure S1), in accordance with other similar reports [53]. The pure hydrogels have temperatures >105 °C in all cases, where their corresponding silver nanocomposites show an important increase in those values, particularly in the case of HPMC hydrogels. For the hydrogels based on CS, this effect is diminished since pure CS-2 and CS-6.92 gels displayed temperatures around 150 °C.

**Figure 4:**
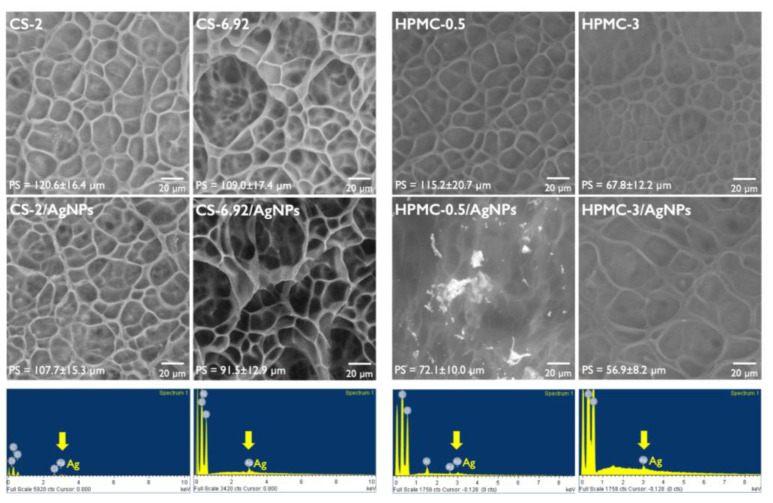
Representative SEM micrographs for pure and silver composite hydrogels. Mean values (PS = pore size) were calculated from three different images per sample. Scale bars = 20 µm in all cases. Bottom: EDS analysis of the silver nanocomposites (silver atoms are pointed to by a yellow arrow).

Next, the interior morphology and the cavity sizes of all prepared nanostructures were examined via cryo-SEM. It is reported that the porosity of the hydrogel architecture depends on the nature of the monomer, polymer concentration, and cross-linking density, among other parameters [54]. Figure 4 demonstrates that the hydrogels have a covalent and cross-linked nature, having different sizes depending upon initial biopolymer concentration. Moreover, the hydrophilic character of organic hydrogels is mediated positively by polysaccharides and can also increase their swelling ratio [45]. In a general view, it was found that the pore is enlarged when the initial concentration of the polymer decreases. For example, pure HPMC-0.5 has an average cavity size of 115.2 ± 20.7 µm compared to HPMC-3 hydrogel (67.8 ± 12.2 µm). In the case of pure CS gels, the difference was limited from 120.6 ± 16.4 µm to 109.0 ± 17.4 µm (CS-2 and CS-6.92, respectively).

The incorporation of silver NPs into the hydrogels affected their average pore size along with their swelling behavior (see Figure 1), probably due to the fact that many functional groups are now interacting with the metallic particles instead of water. Hence, all nanocomposites display a reduction in size compared to the pure CS and HPMC hydrogels. In this sense, the presence of Ag atoms in the embedded NPs into CS-2/AgNPs, CS-6.92/AgNPs, HPMC-0.5/AgNPs, and HPMC-3/AgNPs composites was confirmed by means of EDS elemental analysis (Figure 4 bottom). Complementarily, from the FTIR results, it can be concluded that the high peak intensity observed in CS-2/AgNPs and HPMC-0.5/AgNPs spectra (Figure 3b,d), is related to the amount of metal anchored to the polymeric matrix (level of agglomeration), as detailed in their cryo-SEM images.

**Figure 5:**
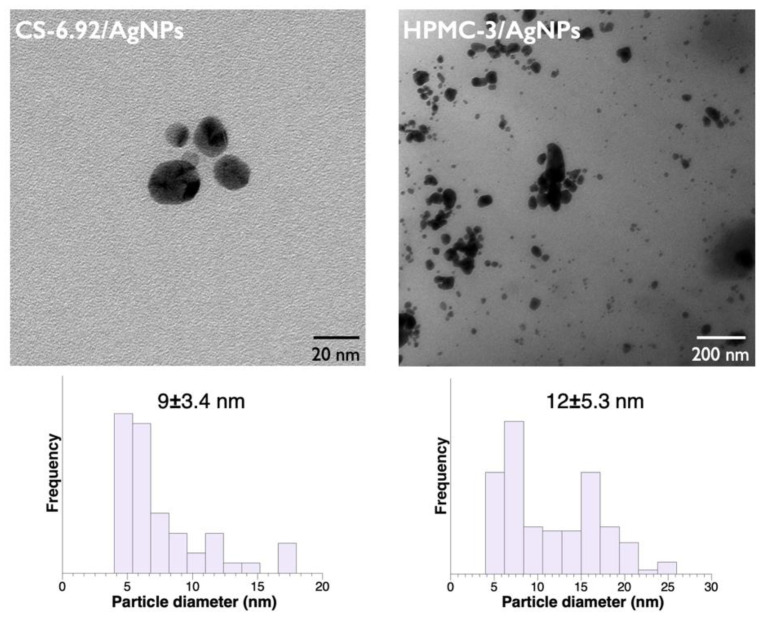
Representative TEM images for CS-6.92/AgNPs and HPMC-3/AgNPs composite hydrogels, including their respective size distribution histograms (bottom). Mean values were calculated from individual particle measurements from different samples. Frequency indicates the relative size of NPs in the population.

Finally, the typical TEM micrographs of the synthesized AgNPs provide essential information about their shape and size distribution. For CS/AgNPs and HPMC/AgNPs hydrogels, these properties are affected by the initial concentration of each biopolymer. Consequently, the smaller particle size is given by a higher polymer concentration, as depicted in Figure 5. The high magnification TEM images reveal the formation of small spherical silver particles, with a narrow size distribution from 4 nm to 18 nm for CS-6.92/AgNPs and diameters about 5–26 nm in the case of HPMC-3/AgNPs, demonstrating the stabilizing effect achieved with the inclusion of polysaccharides into nanostructured hydrogels. When comparing these results with similar others found in literature—other hydrogel species or in aqueous suspension—it is possible to remark on the importance of using polymeric matrices such as CS and HPMC during the stabilization of AgNPs, which directly impacts the obtained particle size, shape, and its distribution (see Table 2).

### 3.4. Evaluation of Antimicrobial Activity

**Figure 6:**
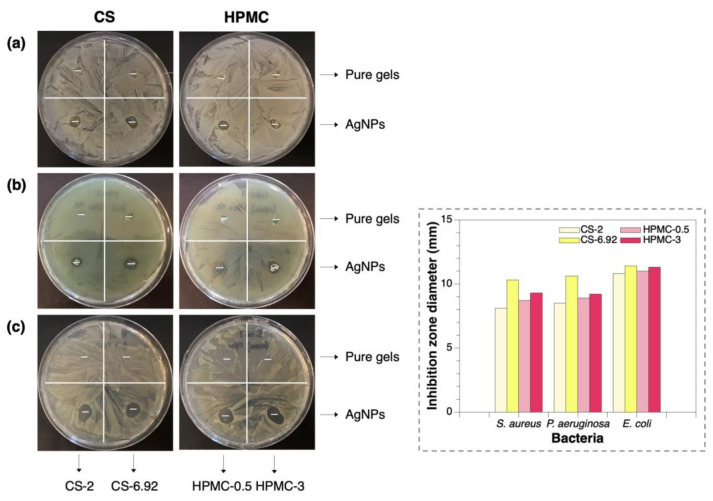
Antibacterial activity of the prepared CS and HPMC-based hydrogels and silver nanocomposites against (**a**) *S. aureus*, (**b**) *P. aeruginosa*, and (**c**) *E. coli*. Pure gels served as controls for all bacteria. Hydrogel codes are at the bottom. Scale bars = 6.0 mm (initial hydrogel size). In the inset (right), the calculated inhibition zones for all groups. All diameters are in mm and averaged from two independent antibacterial assays.

The antibacterial activity of the prepared silver nanocomposites was evaluated against both Gram-negative (*P. aeruginosa*) and Gram-positive bacteria (*S. aureus* and *E. coli*) using the qualitative agar diffusion assay. Representative photographs are illustrated in Figure 6, where all the hydrogels embedding AgNPs displayed a significant inhibition zone (≥ 9.2 mm). This clearly demonstrates a bactericidal effect over the tested bacteria. However, control CS and HPMC hydrogels exhibit a diminished to moderate effect, which might be due to the intrinsic bioactivity of their constituent polymers [18,60]. The calculated inhibition diameters are in agreement with the value reported in the standard control method [61], suggesting that zones >1 mm are labeled as a good antibacterial material. For all tested bacteria, the diameter of inhibition increases at higher polymer concentrations, meaning that among the obtained nanocomposites, CS-6.92/AgNPs and HPMC-3/AgNPs exhibited the most active antibacterial properties and are more effective materials compared to nanoparticles in suspension, improving the durability, stability, mechanical properties, and affinity of the particles during the assay [19,32]. This observation seemed to be due to the small size of the particles found in those samples, as a result of the TEM experiments (Figure 5); therefore, they will have larger surface areas available for interactions, producing a greater bactericidal effect compared to larger NPs [55]. Generally, another important feature that inorganic composite hydrogels offer in biomedical fields is an improvement in the binding characteristic of the NPs towards bacteria—including resistant strains—during the antimicrobial assay [56,62,63,64,65,66].

**Figure 7:**
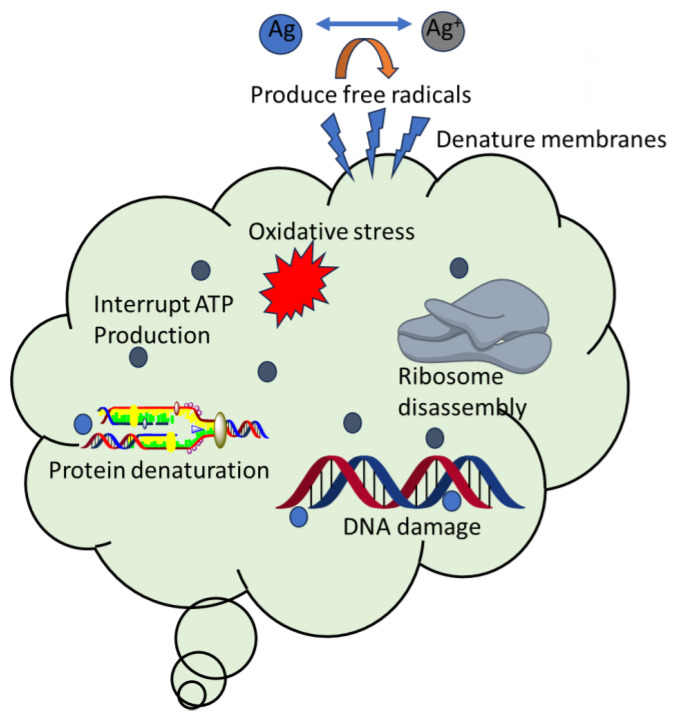
Proposed antibacterial effects of AgNPs over bacterial cells.

Regarding the mechanism of AgNPs, the exact antibacterial effect has not been studied clearly; however, several ideas have been proposed, such as cell membrane breakage, protein denaturation, DNA damage, ribosome disassembly, oxidative stress, and interrupt ATP production [67], as it can be summarized in Figure 7. Moreover, silver nanoparticles are spontaneously liberating Ag ions, and both are having a great affinity towards sulfur, so they can adhere to the bacterial cell wall then which leads to the process of membrane breakage and other above-mentioned effects, avoiding the intrinsic antibiotic resistance mechanisms from resistant strains, which supports the importance of developing new nanomaterials as effective antimicrobial agents in this field.

### 3.5. Theoretical Analysis

**Figure 8:**
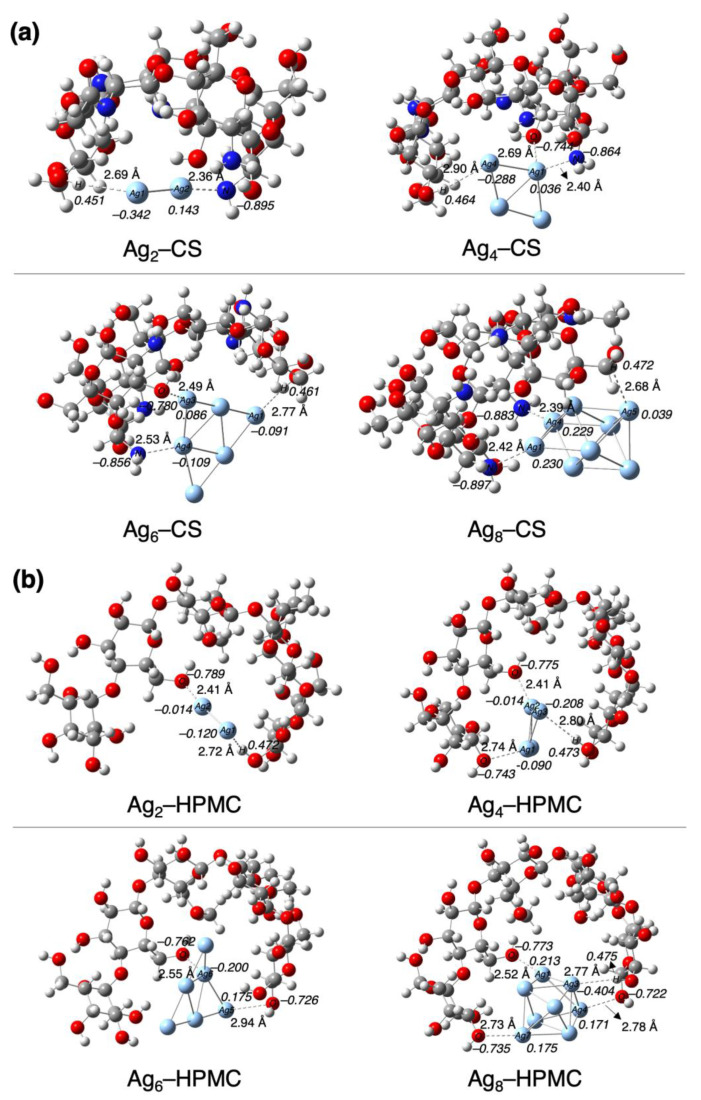
Ground state geometries of the (**a**) Ag_n_–CS and (**b**) Ag_n_–HPMC complexes at the B3LYP/6-311G(d,p)//LANL2DZ level. Bond lengths (Å) and NBO charges (a.u., in italics) for selected atoms are displayed.

DFT calculations delivered information about the electronic properties and binding geometries between selected silver nanoclusters as nanoparticle models [68], Ag_n_ (*n* = 2, 4, 6, and 8 atoms), and the molecular models of both CS and HPMC polymers (depicted in Figures S2 and S3). Also, the analysis provided details of the ionization potential, electron affinity, and charge distribution of the complexes. The optimized Ag_n_–CS and Ag_n_–HPMC complexes are summarized in Figure 8, where the corresponding bond lengths and atomic charges at selected sites involving silver atom interactions are shown. According to the analyzed Ag_n_–CS complexes, it is important to notice that the Ag–N bonds exhibited the lowest distances compared to those shown by silver atoms and the –OH groups, meaning that the nitrogen atoms generate a more stable bonding and have a larger contribution in the stabilization of the studied nanoclusters. In this context, silver atoms can strongly interact, through their 4d and 5s orbitals, with the fully available lone pair of electrons belonging to amine sites. A similar tendency has been reported in investigations of small gold nanoclusters [69]. This theoretical evidence is in good accordance with previous experimental studies, where the coordination of metal clusters by CS is mediated by hydroxyl and amino groups near to the glycosidic linkage [70].

On the other side, the internal coordination between Ag_n_ clusters and HPMC is reinforced through short-medium distance H–bonds. The nature of these interactions helped to elucidate some of the experimental findings about the role of hydroxyl groups, which can strongly interact with silver cations and subsequently stabilize the nanoparticles during their synthesis [16,17]. This approach has been also reported in other cellulose derivatives [71]. Taken together, these results for the Ag_n_–CS and Ag_n_–HPMC complexes established that the geometry of the silver clusters remains almost unchanged.

It is well known that the frontier orbitals analysis comprises a good approach to describing conductivity in molecular systems [69,72]. Thus, the frontier orbitals of the CS and HPMC models and their complexes with silver clusters were examined. The HOMO-LUMO gap was computed as the difference between the highest occupied molecular orbital (HOMO) and the lowest unoccupied molecular orbital (LUMO). As displayed in Table 3, observing the H-L gap energies of the bare optimized polymers, it is clear that once CS and HPMC bind to the silver cluster, the energy gap decreases, indicating greater conductivity. In addition, the complexation energies (∆*E_complexation_*) analysis reveals negative values in all cases, indicating that the complex formation by CS and HPMC is energetically favored, showing minimum energy (see Figure S4a). In detail, the Ag_n_–CS complexes follow the tendency Ag_8_ > Ag_4_ > Ag_2_ > Ag_6_, whereas the Ag_n_–HPMC complexes follow the tendency Ag_4_ > Ag_2_ > Ag_6_ > Ag_8_. Taking the results of the natural bond orbital (NBO) analysis, it is simple to determine that an electron transfer from the polymer to the metal cluster occurred in all complexes, which is associated with the stabilization process. The net charge of the silver cluster in complexation with CS follows an increasing behavior, reaching the most negative value in the Ag_8_–CS complex (*∆q* = −0.483 a.u.), which agrees with its higher stability. Referring to HPMC complexes, the charge values also manifest an ascending trend, where the most negative value is consequently reached in the Ag_8_ cluster (−0.359 a.u.). However, in all silver nanoclusters, the charges are lower in the Ag_n_–CS complexes as a sign of a higher amount of transferred electron density compared to Ag_n_–HPMC complexes (Figure S4b).

Moreover, it is well established that electronic properties like ionization potential (IP) and electron affinity (EA) govern the reactivity of molecular systems. These properties can be estimated through the frontier molecular orbitals energies; according to Koopmans’ theorem [73,74], IP can be approximated as the negative of HOMO energy (*IP ≈ –E_HOMO_*), and EA as the negative of LUMO energy (*EA ≈ –E_LUMO_*). The predicted IP and EA are summarized in Table 3 and graphed in Figure S4c,d. From the data, IP values exhibit an oscillatory behavior depending on the nanocluster size and are in the range of 4.515–5.355 eV for CS and 4.390–5.133 eV for case HPMC, all of them being smaller compared to the bare optimized CS and HPMC structures. Next, the EA values of CS and HPMC models are very low (0.034 eV and 0.021 eV, respectively) in comparison with all the complexes, particularly when the Ag cluster reaches *n* = 4–8 atoms. As the silver nanoclusters have higher IP and EA values than when forming complexes with CS or HPMC, the reactivity increases in these Ag_n_-CS/HPMC systems, suggesting that the formation of metal aggregates is favored.

**Figure 9:**
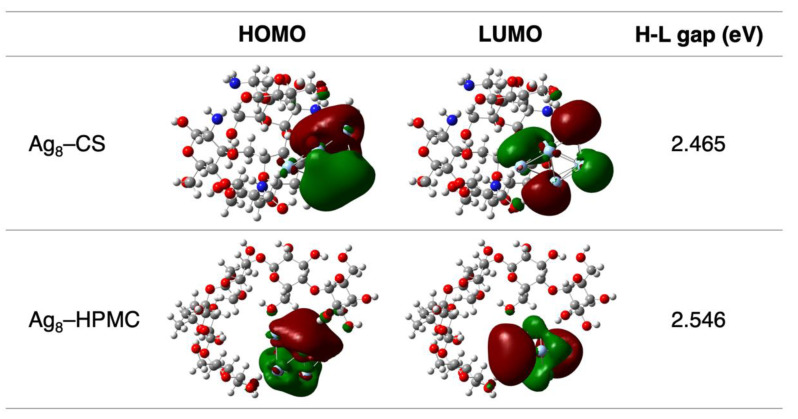
Isosurface plots of the HOMO and LUMO for the Ag_8_–CS and Ag_8_–HPMC complexes and their energy gap.

Finally, chemical bonding in metal complexes is determined by electrostatic and covalent interactions between the components. The covalent contribution is translated during the mixing of molecular orbitals, whereas the electrostatic effect is related to the atomic charges [69,75]. Therefore, Figure 9 displays the shapes of HOMO and LUMO for the biggest silver cluster, Ag_8_, in complexation with CS and HPMC. As can be seen, both frontier orbitals are mainly localized on the silver clusters, suggesting a significant covalent character. The orbital mixing is also reflected in the other studied complexes: Ag_2_–, Ag_4_–, and Ag_6_–CS/HPMC.

## 4. Conclusions

In summary, we have synthesized and characterized hybrid composite hydrogels based on metallic silver nanoparticles and CS and HPMC biopolymers (CS/AgNPs and HPMC/AgNPs) through a simple and flexible two-step methodology. The swelling–deswelling cycles indicate that the developed nanocomposites have good mechanical properties in terms of their structural stability. Moreover, from the cryo-SEM studies, the nanostructures reveal a well-distributed cavity size, where the inclusion of AgNPs improved their thermal stability. The AgNPs deposited into hydrogels are small and spherical in shape, finding that both CS and HPMC polymers have a stabilizing effect, owing to their functional groups (hydroxyl or amino). Through TEM analysis, it was found that the diameters of the NPs were about 9 ± 3.4 nm and 12 ± 5.3 nm for CS-6.92/AgNPs and HPMC-3/AgNPs composites, respectively. The antibacterial assays showed significant inhibition zones against bacteria attributed to the reduced-size AgNPs, demonstrating the great potential of these silver nanocomposites for advanced antimicrobial applications. Despite the achieved results, additional evaluation of the prepared nanomaterials is needed, for instance, to perform cytotoxicity tests or quantitative antibacterial assays. Nevertheless, this method could be extended to other natural polymers and target metals (e.g., gold, copper) in the fabrication of metal composite hydrogels pursuing biomedical or pharmaceutical applications. Finally, theoretical calculations contributed to describe the covalent nature of the interactions between silver nanoclusters and CS and HPMC models, demonstrating that CS has a higher stabilizing effect over the studied complexes.

## Figures and Tables

**Table 1:** The feed composition of the CS and HPMC hydrogels.

Hydrogel	Polymer	AAm (g)	MBA (mM)	TMEDA (mM)	APS (mM)	*S*_g/g_
H-0 (control)	–	1.0	6.48	8.62	21.91	13.2
CS-2	2.0 mg/ml	1.0	6.48	8.62	21.91	18.9
CS-6.92	6.92 mg/ml	1.0	6.48	8.62	21.91	23.2
HPMC-0.5	0.5% (*w/v*)	1.0	6.48	8.62	21.91	21.5
HPMC-3	3.0% (*w/v*)	1.0	6.48	8.62	21.91	22.7

**Table 2:** Comparison with previously reported silver nanoparticles.

Medium, Matrices for NPs	Shape	Reported Size of NPs (nm)	Reference
Aq. suspension, PVP	Spherical	20–80	[5]
Aq. suspension, CS	Cubic	–	[14]
Aq. suspension, HPMC	Spherical	3–17	[16]
Aq. suspension	Cubic	26	[21]
Aq. suspension	Spherical	2–3	[24]
Film, HPMC	–	41–100	[27]
Hydrogel	Cubic	~1 up to 80	[48]
Aq. suspension	Spherical	8.3–14.8	[50]
Hydrogel	Spherical	35–40	[55]
Hydrogel, collagen	Spherical	3.5 ± 0.04	[56]
Hydrogel, curcumin	–	18.24 ± 4.20	[57]
Hydrogel	Spherical	~12	[58]
Hydrogel, starch	Semi-spherical	4–58	[59]

**Table 3:** Total energy (a.u.), complexation energies (∆*E_complexation_*, kcal/mol), net NBO charges on Ag_n_ (∆*q*, a.u.), ionization potential (IP, eV), electron affinity (EA, eV), and the HOMO-LUMO energy gap (H-L gap, eV) for the studied Ag_n_–CS/HPMC systems.

System	Total Energy	∆*E_complexation_*	∆*q* (Ag_n_)	H-L Gap	IP	EA
CS	−3184.405	–	–	6.378	6.411	0.034
Ag_2_–CS	−3476.020	−25.634	−0.199	3.319	5.355	2.037
Ag_4_–CS	−3767.628	−30.421	−0.323	2.311	4.515	2.204
Ag_6_–CS	−4059.253	−19.857	−0.410	2.747	5.023	2.277
Ag_8_–CS	−4350.901	−37.288	−0.483	2.465	4.598	2.134
HPMC	−3363.556	–	–	6.767	6.788	0.021
Ag_2_–HPMC	−3655.173	−26.326	−0.134	3.451	5.133	1.683
Ag_4_–HPMC	−3946.775	−27.435	−0.300	2.327	4.390	2.063
Ag_6_–HPMC	−4238.411	−23.931	−0.317	2.760	5.115	2.355
Ag_8_–HPMC	−4530.029	−22.385	−0.359	2.546	4.540	1.994

## Data Availability

Research data can be obtained on request from the corresponding authors.

## Supplementary Materials

The following supporting information can be downloaded at: https://www.mdpi.com/article/10.3390/polym15163370/s1, Figure S1: Denaturation temperatures (Td, °C) for the pure CS and HPMC hydrogels and its silver nanocomposites measured using DSC; Figure S2: Optimized structures of silver nanoclusters (Ag*_n_*) at the B3LYP/LANL2DZ level of theory, with geometries of one (n = 2), two (n = 4, 6) and three (n = 8) dimension. Bond lengths in Å; Figure S3: Schematic representation of (a) initial and (b) optimized molecular models of chitosan (CS) and hydroxypropyl methylcellulose (HPMC), consisting of 5 monomeric units in each case. Notice how both polymeric chains contract after the optimization of their geometries (B3LYP/6-311G(d,p)). (Color legend: Carbon = gray; Hydrogen = white; Oxygen = red; and Nitrogen = blue); Figure S4: Plots of (a) ∆Ecomplexation, (b) total charge of silver clusters, (c) ionization potential (IP), and (d) electron affinity (EA) of the Agn–CS/HPMC complexes in the function of silver atoms per cluster.
