# Supplementary material for: Biopolymer-Based Composite Hydrogels Embedding Small Silver Nanoparticles for Advanced Antimicrobial Applications: Experimental and Theoretical Insights
Source: Polymers (Basel). 2023 Aug 11;15(16):3370. doi: 10.3390/polym15163370 (PMC10458816; doi:10.3390/polym15163370)
Supplement: Supplementary file 1 [file polymers-15-03370-s001.zip › polymers-2503168-supplementary.pdf]

Supporting Information

Biopolymer-based composite hydrogels embedding small silver nanoparticles for advanced antimicrobial applications. Experimental and theoretical insights

Moises A. Rojas [1], John Amalraj [2,*], and Leonardo S. Santos [1,*]

[1] Laboratory of Asymmetric Synthesis, Instituto de Química de Recursos Naturales, Universidad de Talca, Talca, Chile

[2] Laboratory of Materials Science, Instituto de Química de Recursos Naturales, Universidad de Talca, Talca, Chile

[*] Correspondence: jamalraj@utalca.cl (J.A) and lssantos@utalca.cl (L.S.S)

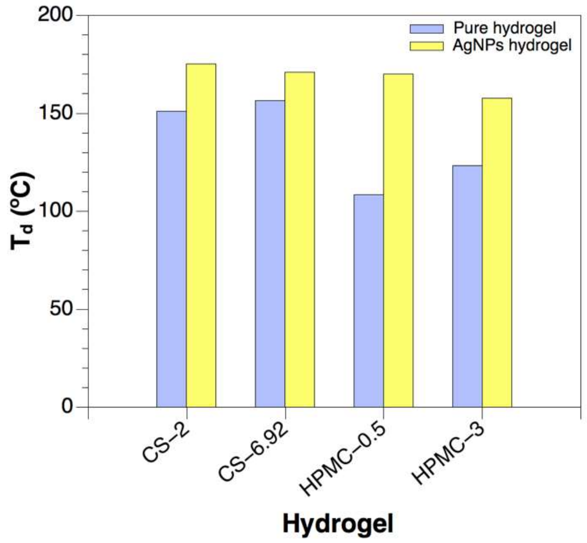

Figure S1. Denaturation temperatures (T_d, ºC) for the pure CS and HPMC hydrogels and their silver nanocomposites were measured using DSC.

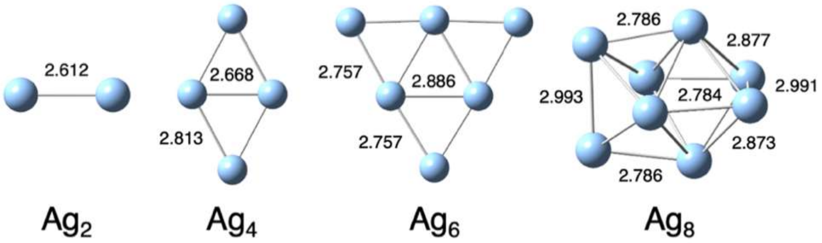

Figure S2. Optimized structures of silver nanoclusters (Ag_n) at the B3LYP/LANL2DZ level of theory, with geometries of one (n = 2), two (n = 4, 6) and three (n = 8) dimension. Bond lengths in Å.

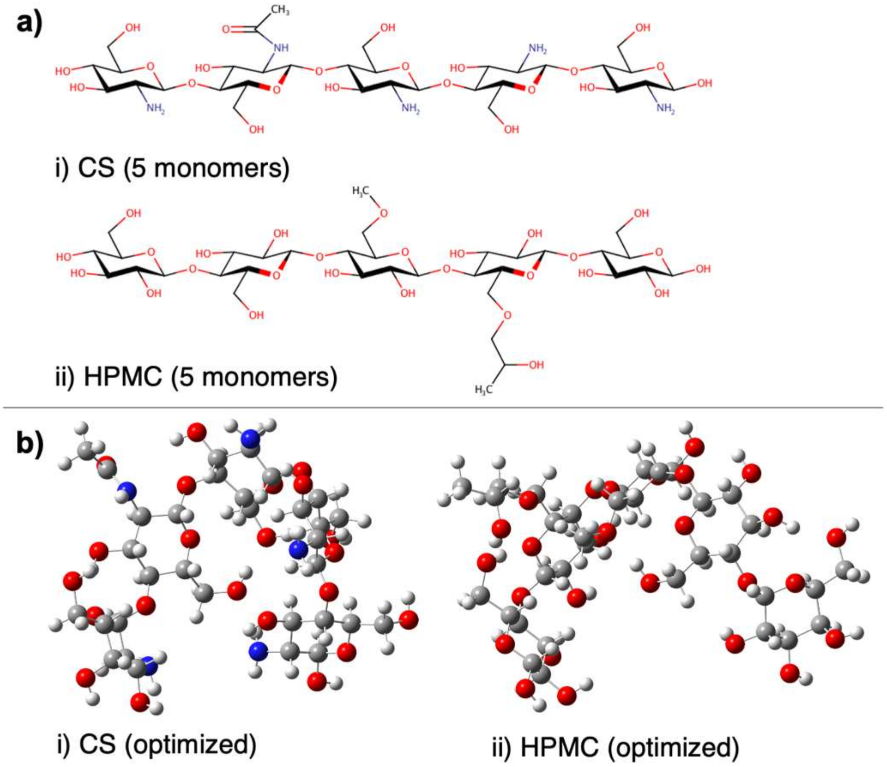

Figure S3. Schematic representation of **a)** initial and **b)** optimized molecular models of chitosan (CS) and hydroxypropyl methylcellulose (HPMC), consisting of 5 monomeric units in each case. Notice how both polymeric chains contract after the optimization of their geometries (B3LYP/6-311G(d,p)). (Color legend: Carbon = gray; Hydrogen = white; Oxygen = red; and Nitrogen = blue).

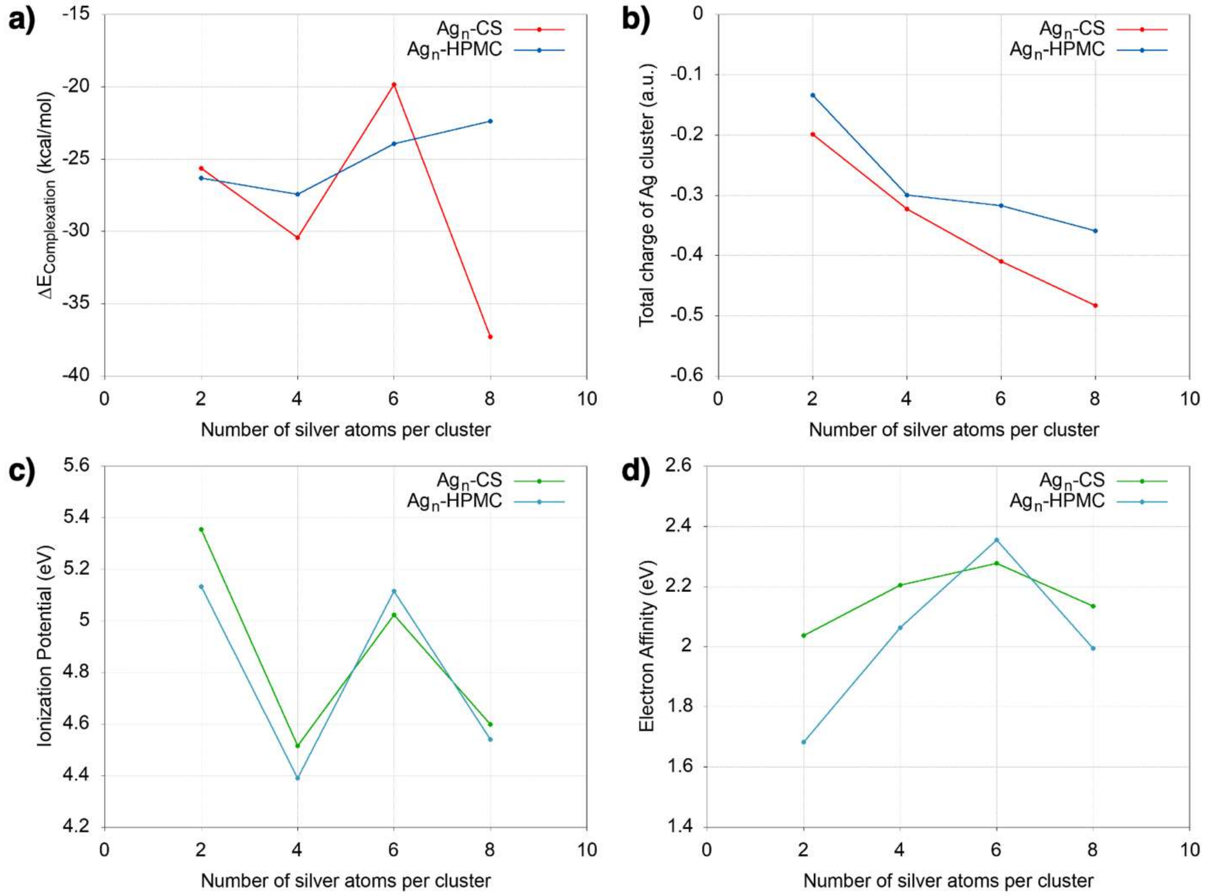

Figure S4. Plots of **a)** $\Delta E_{complexation}$, **b)** total charge of silver clusters, **c)** ionization potential (IP), and **d)** electron affinity (EA) of the Ag_n–CS/HPMC complexes in the function of silver atoms per cluster.
